# The impact of baby schema on perceived attractiveness, beauty, and cuteness in female adults

**DOI:** 10.1186/s40064-015-0940-8

**Published:** 2015-04-07

**Authors:** Kana Kuraguchi, Kosuke Taniguchi, Hiroshi Ashida

**Affiliations:** Department of Psychology, Graduate School of Letters, Kyoto University, Yoshida-honmachi, Sakyo, Kyoto 606-8501 Japan; Research Center for Psychological Science, Doshisha University, Kyoto, Japan

**Keywords:** Attractiveness, Face perception, Beauty, Cuteness, Baby schema

## Abstract

Beauty and cuteness are considered to represent different aspects of attractiveness and to be distinguishable from each other by their respective reliance on neonate and sexually mature features found in attractive faces. In this study, we investigated whether baby schema features in adult faces affect not only cuteness, but also beauty and attractiveness. We also investigated possible differences among attractiveness, beauty, and cuteness, and possible effects of perceived youth on these judgments. Results showed that baby schema features affected judgments of attractiveness, beauty, and cuteness, but that perceived youth did not significantly influence these judgments. Furthermore, the effect of each facial feature differed across rating types with the participants’ naïve interpretation of rating categories. This suggests that beauty predominantly refers to sexual attraction, while attractiveness refers to a non-sexual attraction regardless of participants’ gender. However, gender differences may exist in judging cuteness. Therefore, expressions related to attractiveness may incorporate different elements and this distinction may not be fully shared across gender.

## Introduction

Attractiveness provides observers with useful information for mate selection, but also has various biological benefits. Rhodes (2006) postulated that attractiveness might be categorized into different types, such as sexual attractiveness and cuteness. Among them, the distinction between “beauty” and “cuteness” is typically clearly defined: beauty is considered to reflect averageness, symmetry, and sexual dimorphism (Rhodes 2006), while cuteness is considered to reflect baby schema (Alley 1981; Hildebrandt and Fitzgerald 1979). In other words, beauty is related to sexual maturity (Rhodes 2006), while cuteness refers to the attractiveness of infants (Karraker and Stern 1990).

If beauty and cuteness represent different concepts, the criteria of judging beauty and cuteness should be distinguishable from one another. For example, it is assumed that neonate features are used for judging cuteness, but not for judging beauty. Cunningham (1986), however, showed that attractive female faces possess both neonate and maturity features. Additionally, highly attractive faces have both neonate and sexually dimorphic features (Pfluger et al. 2012). Similar facial features may therefore affect both beauty and cuteness. However, the features that individuals focus on, or the way in which facial features are viewed holistically, may differ for these two types of judgment. Accordingly, it is worth investigating the degree to which there is overlap between the facial features on which both beauty and cuteness depend.

Lorenz (1943) proposed that baby schema is defined by a set of infantile physical features such as a large head, a high and protruding forehead, large eyes, and chubby cheeks, and that these traits create a perception of cuteness that elicits caretaking behavior from adult individuals. Indeed, infant faces are rated as more pleasant and attract more attention than adult faces regardless of participants’ gender (Brosch et al. 2007). Infants with high baby schema are rated as cuter than those with low baby schema regardless of participants’ gender (Glocker et al. 2009). On the other hand, the effects of baby schema may also apply to adult faces. Adult female faces with a configuration of features that appear to be more infantile have been rated as more attractive (Geldart et al. 1999a). Adult faces of males and females manipulated to have infant-like traits have been rated as cuter than the same faces manipulated to have adult-like traits regardless of participants’ gender (Little 2012). Moreover, adult female faces with a high level of baby schema elicit caretaking behavior (Keating et al. 2003). Baby schema therefore affects impression formation not only for infant faces, but also for adult faces. This is supported by the finding that babyfaceness is related to attractiveness (Zebrowitz et al. 1993).

In this study, therefore, we investigated how baby schema features in adult female faces affect judgments of cuteness, attractiveness, and beauty. In still images of Japanese female faces, we measured eye size and the vertical forehead-to-face ratio, both of which have been shown to relate to baby schema (Glocker et al. 2009). A broad forehead is one of the typical facial features found in attractive adult females (Sforza et al. 2009), and adults rate photographed faces with an average-sized or large forehead as more attractive than those with smaller forehead (Geldart et al. 1999a). Large eyes are also found to be an attractive facial feature in adult female faces (Cunningham 1986; Geldart et al. 1999b). In short, these facial features relate both to baby schema and the attractiveness of female faces. Our aim is to reveal how these facial features affect perception of attractiveness, beauty, and cuteness in adult females. We also investigated whether lateral differences in eye-to-face ratios affected participants’ ratings. Franklin and Adams (2010) showed that sexual attractiveness was related to the left visual field, while non-sexual attractiveness was related to the right visual field.

Perceived age is likely to be a possible confounding variable. Mathes et al. (1985) identified a negative relationship between the age and attractiveness of female faces, regardless of the gender or age of the raters. This correlation of attractiveness of female faces with perceived youth has been confirmed by subsequent studies (Tatarunaite et al. 2005) and a relationship between cuteness and infancy has even been found in the faces of children (Volk et al. 2007). Baby schema features in adult faces may affect attractiveness indirectly through perceived youth. We therefore investigated how perceived age affects attractiveness, beauty, and cuteness. Notably however, we only used images of young adults to reduce the effect of actual age, and our discussion on perceived age may not be extended to the general conclusion.

In sum, the purposes of this study are 1) to investigate the effect of baby schema features on judging attractiveness, beauty, and cuteness, and 2) to investigate whether perceived youth affects these judgments. We hypothesized that baby schema features might affect the judgments of attractiveness, beauty, and cuteness in different ways, as regards to the lateral difference of the eyes and the effect of age.

## Methods

### Participants

The participants in this study were 69 undergraduate and graduate students (33 men, 36 women, aged from 18 to 25). We obtained informed written consent from all participants, and each was paid according to the standards of Kyoto University. All participants had normal or corrected-to-normal vision.

### Stimuli

Facial photographs of 17 Japanese female students aged from 18 to 24 were used. The faces in all images held a neutral expression and were centrally positioned. The all models were recruited from other universities in Kyoto, and were not acquainted with the participants. We obtained written informed consent from all those photographed, and these individuals were paid according to the standards of Kyoto University. In order to eliminate the effect of hair-style, we cut the images into an ellipse roughly along the facial outline. To reduce any effect of skin color on judgments, we converted the images to gray scale. These manipulations were conducted by using Photoshop 5.5 (Adobe Systems).

### Procedure

We presented the stimuli one by one for 2 s in random order on a 19-inch CRT monitor (Sony). Participants were then asked to make a judgment on each picture by choosing a value on a scale ranging from 1 to 6 (e.g., for the cuteness judgment, 1 = *not cute* and 6 = *cute*) by pushing the corresponding key after the stimulus disappeared. Judgments were solicited on “attractiveness,” “beauty,” “cuteness,” and “age”. Judgments were collected in separate blocks, and the order of these blocks was randomized for each participant. Participants were asked to guess the age of the photographed individual and to answer the estimated age in years after the other judgments in order to reduce any possible age bias in favor of younger individuals. SuperLab 4.5 for Windows (Cedrus, Inc) was used to control the experiment.

### Measurements of facial features

**Figure 1:**
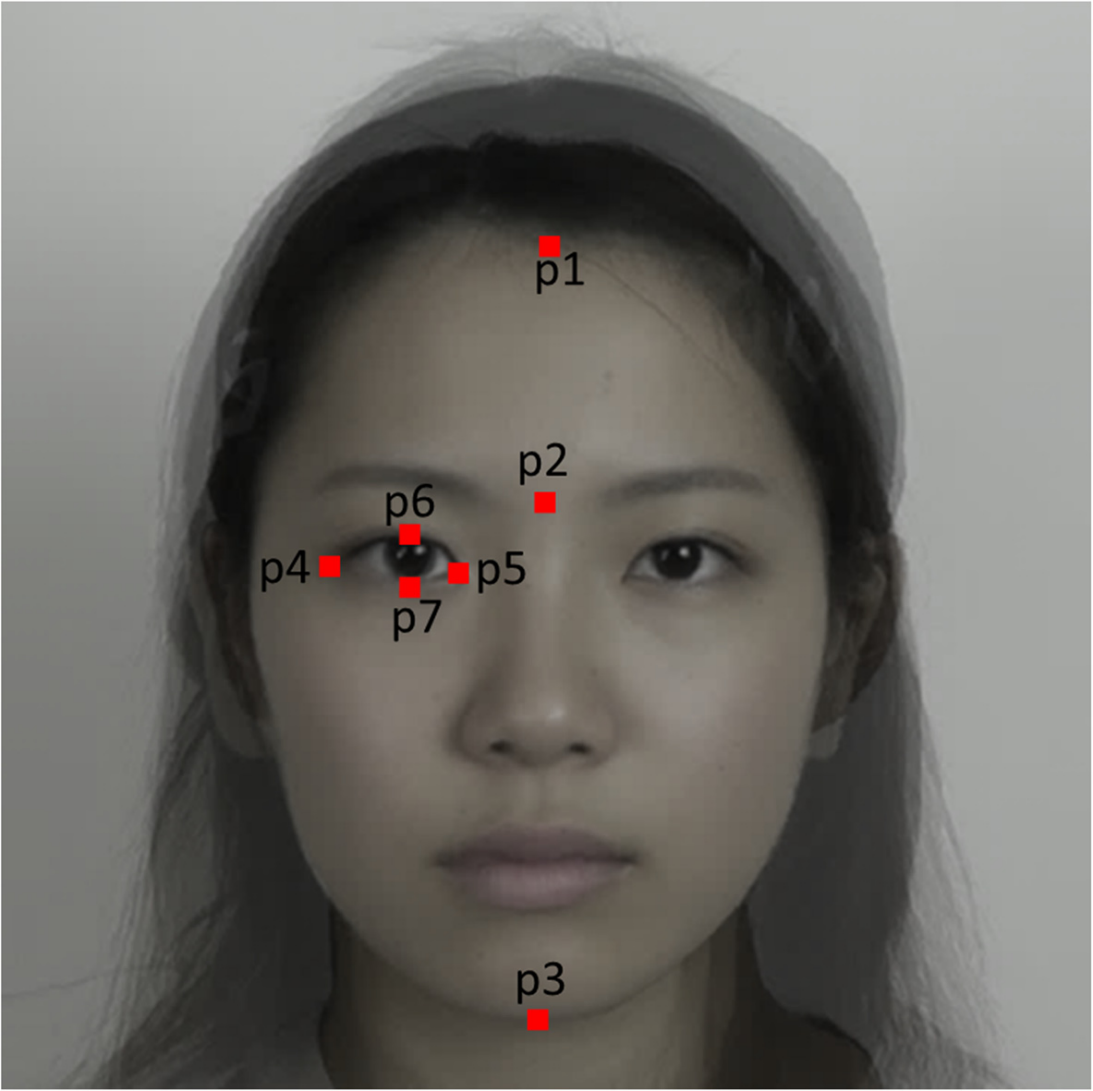
**Facial landmarks for measurement.** We calculated (the distance between p1 and p2)/(the distance between p1 and p3) for the vertical forehead-to-face ratio, (the distance between p6 and p7)/(the distance between p1 and p3) for the vertical eye-to-face ratio, and (the distance between p6 and p7)/(the distance between p4 and p5) for the roundness of the eye. Eye size was calculated separately for the right and the left eye.

We measured 1) the vertical forehead-to-face ratio and 2) eye size, both of which are related to baby schema. Eye size measurements included both the vertical eye-to-face ratio and the roundness of the eye. Facial measurements were conducted by measuring the distances between facial landmarks (Figure 1) using Photoshop’s measure tool (Adobe Systems). Facial landmarks were determined by referring to the landmarks used in Zebrowitz et al. (2003). Figure 1 shows an averaged face for the purpose of illustration, but each individual image was used for the actual experiment and measurements. Pixels were the unit of measurement. Note that left and right eyes refer to the eyes on the image from the participant’s point of view, not the left and right eyes of the models.

## Results

### Descriptive statistics

The results of facial measurement and rated scores of judgments are summarized in Table 1. Every judgment ranged substantially from low to high ratings, which confirms that the facial stimuli in this study were appropriate to investigate the relationship between facial features and each judgment.

**Table 1:** **Summary of the results**

	**Max**	**Min**	**Average (SD)**
**Facial measurements**			
The forehead-to-face ratio	0.377	0.242	0.296 (0.026)
The *right* vertical eye-to-face ratio	0.072	0.049	0.058 (0.006)
The *left* vertical eye-to-face ratio	0.071	0.047	0.058 (0.005)
The roundness of the *right* eye	0.476	0.331	0.398 (0.035)
The roundness of the *left* eye	0.445	0.323	0.390 (0.030)
**Rated scores (female participants)**			
Attractiveness	4.45	2.20	3.19 (0.749)
Beauty	4.55	2.00	3.24 (0.780)
Cuteness	4.80	1.90	3.25 (0.871)
Age	30.6	18.3	25.18 (3.03)
**Rated scores (male participants)**			
Attractiveness	4.60	2.10	3.12 (0.814)
Beauty	4.55	2.25	3.08 (0.784)
Cuteness	4.45	1.85	2.92 (0.813)
Age	27.5	20.5	23.61 (1.99)

### Structural equation modeling

In order to investigate the causal relationship between facial features and each judgment, we analyzed the data by applying structural equation modeling (SEM), using the sem() function of R language.

**Figure 2:**
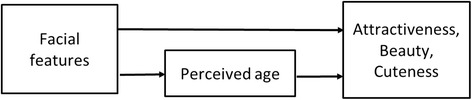
**The initial model.** One-sided arrows represent causal relationships.

**Figure 3:**
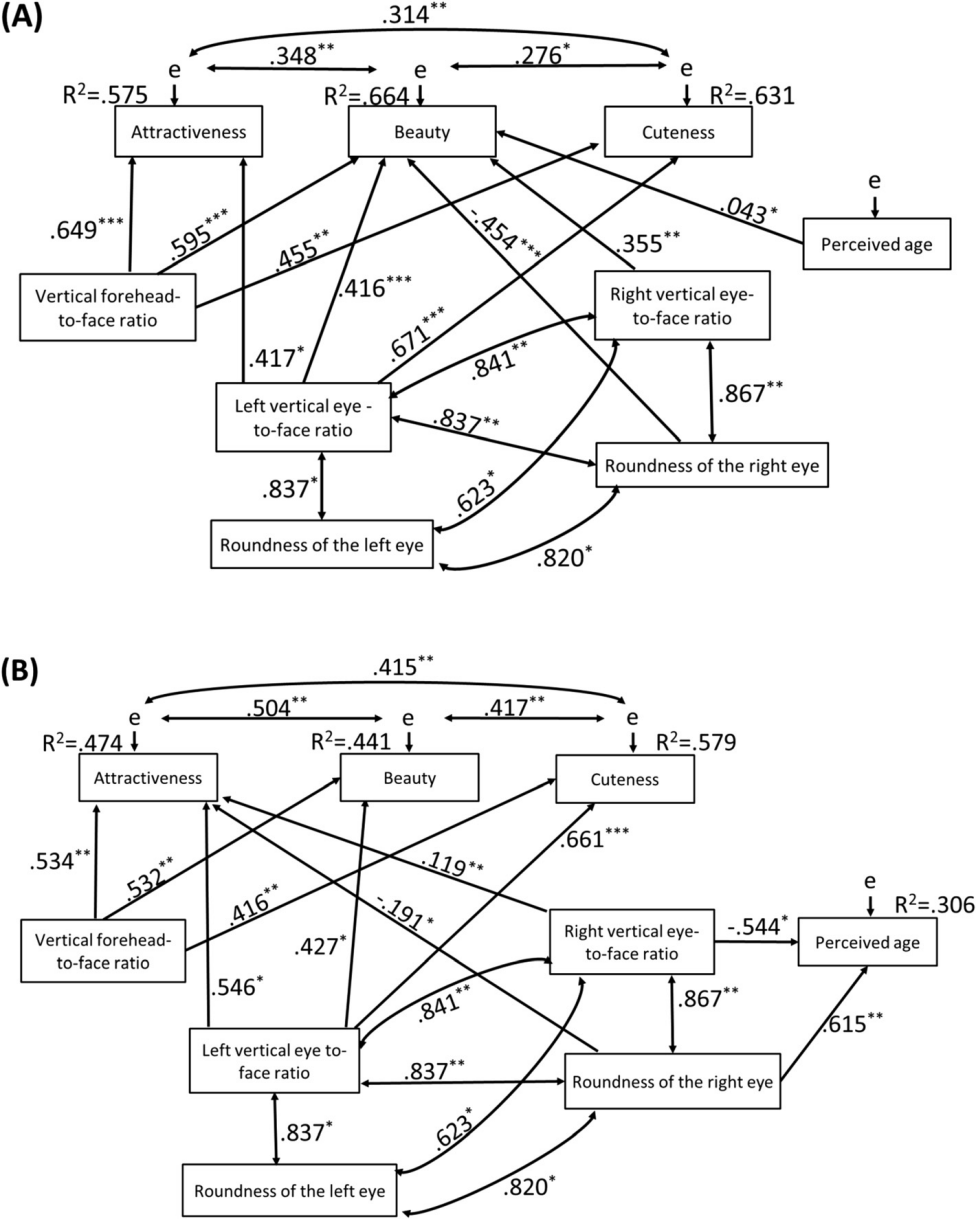
**SEM path diagrams for the combined data set.** Panel **(A)** is for female participants, and panel **(B)** is for male participants. The estimated associations represented by one-sided arrows representing partial regression coefficients and by double-sided arrows representing the partial correlations. The path coefficient values are shown on the arrows, with the significance levels highlighted as follows: * (*p* < .05), ** (*p* < .01), and *** (*p* < .001).

First, we investigated the initial model (Figure 2) that addressed whether facial features and perceived age affected the ratings (beauty, cuteness, and attractiveness). This model was run separately for each gender group of participants. The results are summarized in Figure 3 (females *χ*^2^(16) = 7.63, *p* = .958, GFI = .913, NFI = .963, CFI = 1.00, RMSEA = 0.00, AIC = 65.63; males *χ*^2^(15) = 7.49, *p* = .942, GFI = .915, NFI = .964, CFI = 1.00, RMSEA = 0.00, AIC = 67.49). Both models demonstrated good explanatory power and significant correlations were found among attractiveness, beauty, and cuteness regardless of participants’ gender. Further, attractiveness, beauty, and cuteness were positively influenced by both the vertical forehead-to-face ratio and the left vertical eye-to-face ratio. In addition, for female participants, beauty was positively affected by the right vertical eye-to-face ratio, but negatively affected by the roundness of the right eye. For male participants, attractiveness was positively affected by the right vertical eye-to-face ratio, but negatively affected by the roundness of the right eye.

As strong correlations were found among attractiveness, beauty, and cuteness, each rating might conceivably have affected the other ratings throughout this experiment. In order to reduce the effect of such interactions, we conducted SEM with ratings of the first block only for each gender. This model did not show a good fit for female participants with a GFI below .90 (*χ*^2^(17) = 13.38, *p* = .709, GFI = .863, NFI = .927, CFI = 1.00, RMSEA = 0.00, AIC = 69.38). We therefore removed the effect of perceived age on the ratings from the initial model for female participants’ data, and re-conducted the SEM.

**Figure 4:**
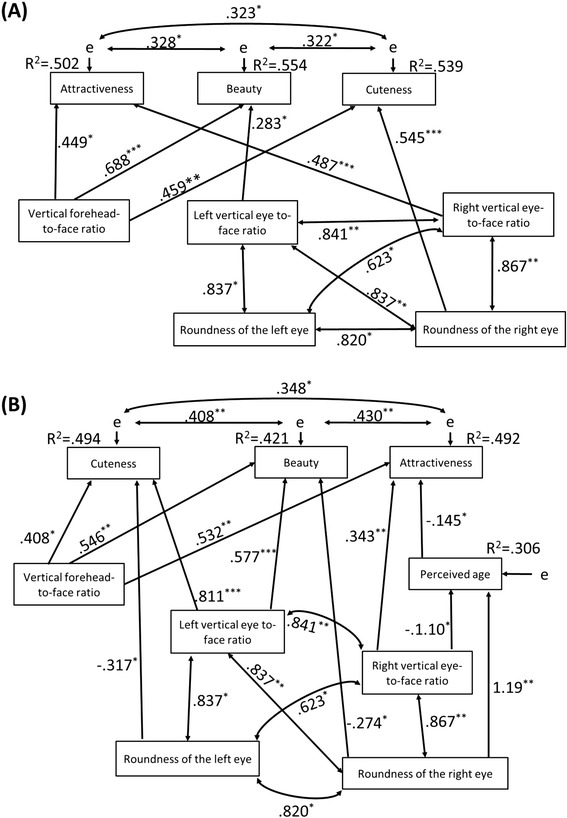
**SEM path diagram for the initial block data.** Panel **(A)** is for female participants, and panel **(B)** is for male participants.

The final models are summarized in Figure 4 (females *χ*^2^(11) = 8.43, *p* = .673, GFI = .900, NFI = .951, CFI = 1.00, RMSEA = 0.00, AIC = 58.43; males *χ*^2^(14) = 6.31, *p* = .957, GFI = .926, NFI = .966, CFI = 1.00, RMSEA = 0.00, AIC = 68.31). These models have good explanatory power. For both participants’ gender, vertical forehead-to-face ratio affected attractiveness, beauty, and cuteness, and the left vertical eye-to-face ratio affected beauty. Significant correlations were also found among attractiveness, beauty, and cuteness. These results were consistent with models for the combined data set. For the model related to male participants, the left vertical eye-to-face ratio affected cuteness, and the right vertical eye-to-face ratio affected attractiveness. These effects were also consistent with models for the combined data set (Figure 3).

On the other hand, differences were found between the first-block and combined data models. For the female participants’ models, the roundness of the right eye affected cuteness in the first-block model, but beauty in the combined data model, and the right vertical eye-to-face ratio affected attractiveness in the first-block model, but beauty in the combined data model. For the male participants’ models, the roundness of the right eye affected beauty in the first-block model, but attractiveness in the combined data model, and the roundness of the left eye negatively affected cuteness in the first-block model. No effect of the left vertical eye-to-face ratio on attractiveness was found. Perceived age negatively affected attractiveness.

## Discussion

### Causal relationship between facial features and attractiveness, beauty, and cuteness

The facial features measured in this study affected participants’ ratings of attractiveness, beauty, and cuteness. This influence was also found in the initial block data that was supposed to be relatively free from interactions among these ratings. Luo et al. (2011) showed that facial likability and attractiveness were no longer affected by baby schema after 4.5 years of age, but the present study found that ratings of attractiveness, beauty, and cuteness were related to both baby schema features even for adult faces. The vertical forehead-to-face ratio in particular positively affected each rating, an effect that was confirmed regardless of participants’ gender or the order in which judgments were made. This supported the findings of Geldart et al. (1999a). In light of the correlation among the judgments used in this experiment, the concepts may at least partially overlap. However, the facial features related to these judgments were not completely interchangeable. Indeed, baby schema affected all three, but the effect of eye region was different for each judgment type.

The left vertical eye-to-face ratio positively affected beauty regardless of participants’ gender or the order in which ratings were made. We therefore discuss that the perception of the left side of faces from the point of view of the observer may be more important in making judgments on beauty than the right. In previous research, beauty has shown to provide beneficial information to observers, including health status and gene quality for the purposes of mate selection. This is also related to sexual attractiveness (see Rhodes 2006 for a review), a judgment that is associated with the left visual field (right hemisphere; Franklin and Adams 2010). In short, the result that the left eye affected perception of beauty is supported by the finding of previous research (Franklin and Adams 2010), which indicates that participants consider beauty to be an important component of sexual attractiveness even in the absence of explicit definitions.

Cuteness is considered to be a form of non-sexual attractiveness that elicits caretaking behavior (Keating et al. 2003) and motivates social commitment (Sherman and Haidt 2011). Franklin and Adams (2010) showed that non-sexual attractiveness was more associated with the right visual field (left hemisphere). Accordingly, it is expected that cuteness could be more affected by the right eye. We found, however, a difference between genders in this regard. For *female* participants, while the roundness of *right* eye affected cuteness in the first-block model, the *left* vertical eye-to-face ratio affected cuteness in the combined data model. This effect of the left eye may have been a result of confusion with beauty judgments. For male participants, the *left* vertical eye-to-face ratio positively affected cuteness as well as beauty both in the first-block and in the combined data. This may indicate that males always confuse cuteness with beauty, but given the results of the first block that beauty was negatively affected by the roundness of the *right* eye while cuteness was negatively affected by the roundness of the *left* eye, it is more likely that males distinguish between beauty and cuteness. Instead, it is suggested that males might take cuteness of female faces as one aspect of sexual attractiveness. The gender difference in cuteness is generally consistent with previous findings that have found gender differences in the observation of cuteness in infant faces (Sprengelmeyer et al. 2009; Lobmaier et al. 2010).

Attractiveness may incorporate a non-sexual element. For female participants, the right vertical eye-to-face ratio affected attractiveness in the first-block model. Right side information may be an advantage for judging attractiveness, but not beauty. This is also consistent with the finding that the right vertical eye-to-face ratio affected judgments of attractiveness among male participants, while the left vertical eye-to-face ratio affected beauty. It may therefore be suggested that participants distinguish between beauty and attractiveness. Accordingly, when asked solely about attractiveness, non-sexual attraction may be easily estimated.

We should note that the concept of cuteness in Japanese (“kawaii”) might be notably different from that in other cultures in that the Japanese concept may confuse cuteness with beauty to a greater degree (Daibo 2007). The Japanese word for beautiful (“Utsukushii”) originally had a meaning that is closer to “kawaii” in modern Japanese, which may be one of the reasons why we confuse cuteness with beauty. The concept of cuteness is rooted in Japanese traditional aesthetics that cherished small things and found beauty in them (Okayama and Ricatti 2008), and then cuteness may share the meaning with beauty. It is, however, evident that cuteness does not completely overlap with beauty or attractiveness, but was found to be quite distinct in light of the effect of lateral difference of the eye in this study.

Japanese animation or cartoons often have characters with extraordinary large and round eyes. This may be caused by the Japanese culture that valued such a feature positively. Our research indeed showed that the eye size affected the judgments of attractiveness, beauty, or cuteness. However, we also had a result that roundness of the eye is negatively related to some judgments. As Seyama and Nagayama (2007) showed that the big eyes are felt uncannier for real than artificial faces, further research is needed to investigate how preferences to real faces are reflected in cartoon characters.

### The effect of perceived age in similar-age groups

Before discussing the effect of perceived age, it should be noted that we used facial images depicting portraits of a limited age range (18–24 years). We used this limited range in order to have them correspond to the ages of participants (18–25 years) who were university students and would therefore be more likely to have friends or partners within this age brackets. As described later, the effect of age was generally weak in this study, but this is not necessarily contradictory to the results of previous studies that investigated a wider range of facial images. For example, Mathes et al. (1985) used facial images aged from 10 to 70 and over, and Tatarunaite et al. (2005) used faces aged 11 and 31, with both reporting comparable findings.

Perceived age did not affect cuteness in our study, possibly due to the limited age group used, although eye size and forehead-to-face ratio did directly affect this judgment. This is inconsistent with the findings of Volk et al. (2007) who demonstrated a relationship between cuteness and age for children’s faces, but this could be due to the differences in age between the target faces and the participants. Our result indicates that the assessment of cuteness in adults may be independently rated, but it is not always related to infancy or perceived youth, and that cuteness in adult faces is affected mainly by morphological features rather than by perceived youth or infancy, even though such features could be analogous to those used to assess the cuteness of infants.

When male participants judged attractiveness in the first block, perceived age negatively affected attractiveness. This supports the findings of Tatarunaite et al. (2005) that attractiveness is related to perceived youth. This effect, however, was weak and not robust in our study, which could again be due to the limited age range. Furthermore, perceived age did not affect beauty among male participants either. These results indicate that baby schema features may affect beauty, but that this appears to be a direct effect with no indirect influence asserted through the perceived age of target.

For female participants, perceived age may enhance the perceived beauty of female faces, which is not consistent with the results for males. While it is not likely that this effect arouse through confusion with cuteness, the effect was small was not evident in the first-block data. Further studies will have to investigate this possibility, but this effect of age for participant-target pairings of the same gender could be intuitively understood.

Finally, facial features related to the right eye affected perceived age for male participants, but not for female participants. Baby schema features may affect perceived age only for male participants in a similar age bracket to the target individual. Age judgments may depend more on other features, such as skin condition and the presence of wrinkles (Fink and Matts 2008; Fink et al. 2011).

## Conclusion

We found that baby schema features affect judgments of attractiveness, beauty, and cuteness in different ways among young Japanese adults. These three perceptions should be viewed as distinct even without the provision of explicit instructions or definitions, although they might be somewhat confused or intermixed. The results suggest that beauty predominantly refers to sexual attraction while attractiveness refers to a non-sexual attraction regardless of participants’ gender. On the other hand, an association between cuteness and baby schema features was found, although there were gender differences for this effect; cuteness implies non-sexual attraction for female participants, while it implies both sexual and non-sexual attraction for male participants. A limited effect of perceived age was found. These findings suggest that the terms related to attractiveness may incorporate different elements, and that this distinction may not be fully shared across gender.
